# Indication for spinal surgery: associated factors and regional differences in Germany

**DOI:** 10.1186/s12913-022-08492-3

**Published:** 2022-09-01

**Authors:** Falko Tesch, Toni Lange, Patrik Dröge, Christian Günster, Johannes Flechtenmacher, Burkhard Lembeck, Bernd Kladny, Dieter Christian Wirtz, Fritz-Uwe Niethard, Jochen Schmitt

**Affiliations:** 1 Center for Evidence-Based Healthcare, University Hospital and Faculty of Medicine Carl Gustav Carus, TU Dresden, 01307 Dresden, Germany; 2 Allgemeine Ortskrankenkasse (AOK) Research Institute, Berlin, Germany; 3 German Professional Association for Orthopedics and Trauma (BVOU), Berlin, Germany; 4 m&i Fachklinik Herzogenaurach, Herzogenaurach, Germany; 5 German Society for Orthopedics and Trauma (DGOU), Berlin, Germany; 6 Department of Orthopaedics and Traumatology, University Hospital Bonn, Bonn, Germany

**Keywords:** Spinal surgery, Back pain, Health service research, Secondary data analysis

## Abstract

**Background:**

Rising surgery rates have raised questions about the indications for spinal surgery. The study investigated patient-level and regional factors associated with spinal surgery for patients with spinal diseases.

**Methods:**

We undertook a cohort study based on routine healthcare data from Germany of 18.4 million patients within 60.9 million episodes of two patient-years before a possible spinal surgery in the time period 2008 to 2016. Using a Poisson model, the effects of a broad range of patient-related (sociodemographic, morbidity, social status), disease- and healthcare-related (physicians’ specialty, conservative treatments) and regional variables were analyzed.

**Results:**

There was substantial regional heterogeneity in the occurrence of spinal surgery which decreased by only one quarter when controlling for the various determinants assessed. Previous musculoskeletal and mental health disorders as well as physical therapy were associated with a lower probability of surgery in the fully-adjusted model. Prescriptions for pain medication and consultations of specialists were associated with a higher probability of surgery. However, the specific severity of the vertebral diseases could not be taken into account in the analysis. Furthermore, a substantial proportion of patients with surgery did not receive a consultation with an outpatient specialist (29.5%), preoperative diagnostics (37.0%) or physical therapy (48.3%) before hospital admission.

**Conclusion:**

This large study on spinal diseases in Germany highlights important patterns in medical care of spinal diseases and their association with the probability of spinal surgery. However, only a relatively small proportion of the regional heterogeneity in spinal surgery could be explained by the extensive consideration of confounders, which suggests the relevance of other unmeasured factors like physicians’ preferences.

**Supplementary Information:**

The online version contains supplementary material available at 10.1186/s12913-022-08492-3.

## Background

Spinal diseases are common, and often associated with back pain. Back pain is estimated to account for 10.7% of all years lived with disability in Germany [1]. The spectrum of spinal diseases ranges from fractures, degenerative deformities of the spine to inflammatory diseases. For Patients, a wide variety of conservative treatment options (pharmacological/ non-pharmacological) as well as surgeries exists.

The number of spinal surgeries in Germany increased in recent years as well as in other developed countries [2–4]. At the same time significant regional differences in surgery rates for the spine were observed in Germany, the USA and Finland [5–7],while only minor regional differences were observed in Norway and Sweden [8, 9]. In addition, a shift from lumbar disc herniation surgeries towards decompression and fusion surgeries were observed [6, 7]. Furthermore, the per-capita supply of orthopedic surgeons and neurosurgeons were not associated with spinal surgery rates [3]. Regional differences for invasive procedures are also found for knee and hip joint replacement. Previous studies evaluating the indication for these surgeries were either survey based and therefore unable to investigate regional differences or based on aggregated data and thus unable to investigate individual patient factors [10, 11]. For a better understanding of the likely reasons for the regional differences, analyses at the patient level clustered within region are necessary. Such analyses can consider patient and disease specific sociodemographic factors and factors capturing patient-specific utilization of medical care services in order to clarify regional differences in care, in addition to region specific factors.

The aims of the present study were to analyze the association of sociodemographic, morbidity, and also healthcare-related variables with spinal surgeries and to contribute to the elucidation of the observed regional heterogeneity in spinal surgery in Germany.

## Methods

### Data base and study design

The study is based on extensive routine healthcare data collected by the “Allgemeine Ortskrankenkassen” (AOK) and was conducted in accordance with the Good Practice Secondary Data Analysis of the German Society for Epidemiology [12]. In Germany, 90% of the population are members of a statutory health insurance program, of which the AOK is the largest. The AOK represented about 24 million people in the time period 2006 to 2016. The study cohort consisted of insured persons with a prevalent spinal disease according to the 10th revision of the International Statistical Classification of Diseases and Related Health Problems (ICD-10) diagnoses M40-M54 in the outpatient or inpatient sector residing in Germany. The area of Germany was divided by 96 spatial planning regions formed by the German Federal Office for Building and Regional Planning (BBR) rather than the political borders of 16 federal states or 401 counties. Patients were excluded if they had a concomitant fracture of the spine (ICD-10: S12, S22, S32) or less than 350 insurance days per year (patients who died during the year were still included). In the case of surgery, patient characteristics of the outpatient care from the two years prior surgery were used. For patients without surgery, an equivalent two-year calendar period was utilized. A lumbar spine surgery was defined using the respective German operation and procedure code (OPS) (Additional file 1).

For certain population group’s additional information was available in German routine health care data. The cohort was therefore divided into the subgroups of patients over 64 years of age without employment ("retired") and those with employment and an age between 20 and 64 years ("employed"). The analysis in the “employed” group was restricted to the years 2012 to 2016, because a new classification of occupations was introduced in Germany in the year 2011.

### Associated factors

Sociodemographic, morbidity and medical care variables along with calendar year and region were utilized as explanatory factors (Additional files 2, 3 and 4). These variables were selected in consultation with the scientific advisory board of the DEWI project. The aim was to select all relevant diagnostics, conservative treatments, physician’s specialties and those comorbidities relevant to the treating physician. The fundamental difficulty in capturing the effect of therapies in a non-randomized setting is that explanatory factors on the one hand indicate the severity of the patient's disease and on the other hand may also influence the probability for surgery. Therefore, variables from different domains were used in the model to approximate the severity of the spinal disease and so to isolate the therapy effect (Additional file 5).

### Statistical analysis

The plot of surgery rates per patient over time were age-standardized using the 2013 European standard [13]. Poisson regression models were used for multivariable-adjusted relative risks (RR) with 95% confidence intervals (95% CI). Forest plots and a map of Germany were used to graphically display the results. Spatial heterogeneity was captured by the Morans I geographic index [14]. This measure has a range from -1 to + 1. A value of 0 indicates that there is no difference between the regions and a value closer of 1 indicates that spinal surgeries successively decrease with increasing distance from a certain region “positive autocorrelation”. A value below 0 indicates that neighboring regions are more dissimilar in the indicator in focus than more distant ones “negative autocorrelation”. Only first-order spatial lags of the measure were used. Spatial heterogeneity analysis was first performed adjusting for age groups only (base model) and then adjusting for all explanatory variables described (fully adjusted model). Statistical modeling was performed using the R package speedglm version 0.3–2 in the statistical software R [15].

## Results

### Cohort description

**Fig. 1:**
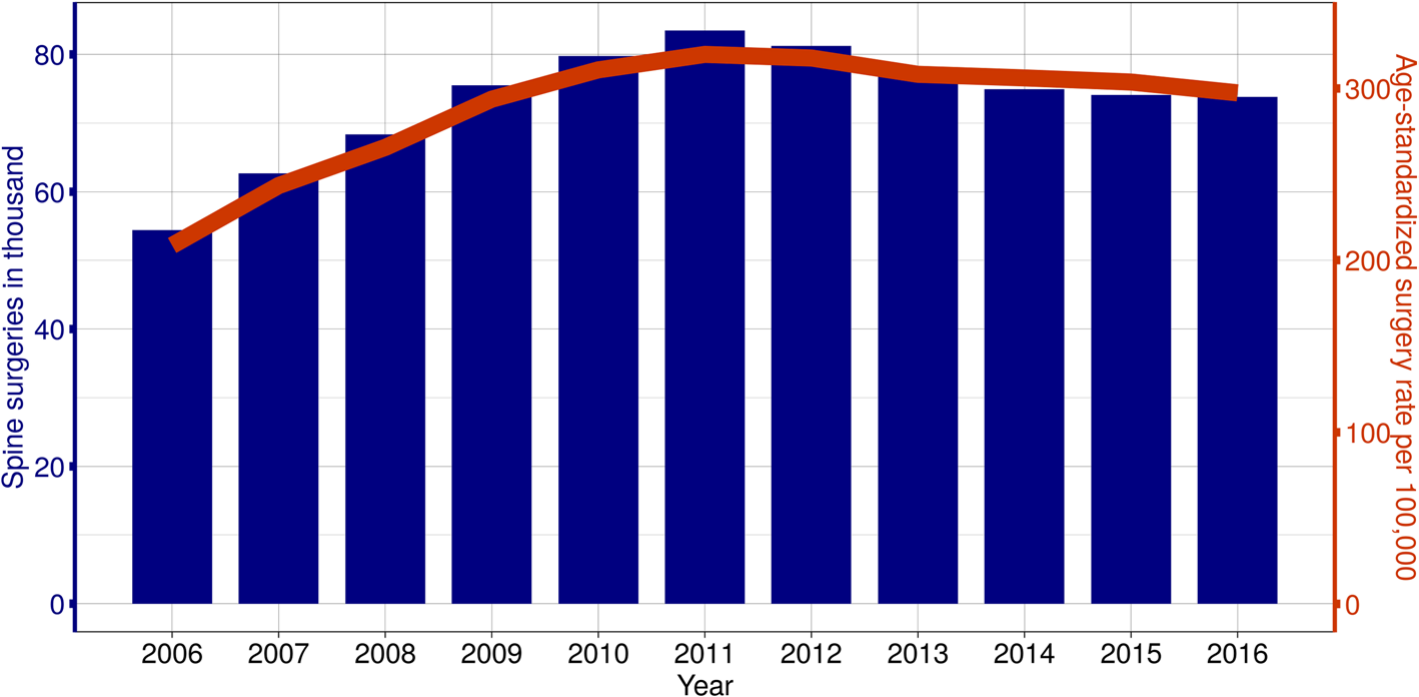
Development over time of spinal interventions in Germany (AOK) with diagnosis ICD-10: M40-M54 in the period 2006 to 2016. The absolute (blue) and relative (orange) surgery rates are shown

The rate of invasive spinal surgeries shows clear regional patterns in Germany with fewer surgeries in the northeast and southwest of Germany. Regional clustering, as measured by Morans I, increased from 0.31 in 2006 to 0.46 in 2016. At the same time, the surgery rate increased considerably in the study population from 208.7 in 2006 to 320.0 per 100,000 insured persons by 2011, then stabilized at about 300 per 100,000 insured persons between 2014 and 2016. In absolute numbers, this was an increase from 54 to 83 thousand patients per year with spinal surgery (Fig. 1). The majority of these patients had a diagnosis of ICD-10: M40-M54 (2016: 69,876 of the 73,820 patients or 94.7%). A total of 18.4 million patients (59% female) with spinal disease in 60.9 million two-year episodes with 444,218 spinal surgeries were included in the analysis. Their characteristics can be found in Table 1 and Additional file 6.

**Table 1:** Sociodemographic, clinical, and medical care characteristics of patient episodes with spinal disease and spinal surgery

	With Spinal Disease		With Spinal Surgery	
	Episodes	Percent	Episodes	Percent
***Sociodemographic***				
Total	60942755	100	444218	100
Male	24839868	40.8	195082	43.9
Female	36102887	59.2	249136	56.1
Age group 0–39	10746004	17.6	34281	7.7
Age group 40–64	26563110	43.6	182757	41.1
Age group 65 +	23633641	38.8	227180	51.1
***Comorbidities***				
Osteoarthritis (knee)	6647788	10.9	5622	12.5
Osteoarthritis (hip)	10616636	17.4	81399	18.3
Osteoporosis	6259585	10.3	54990	12.4
Chronic rheumatoid polyarthritis	1937602	3.2	9579	4.4
Other rheumatic diseases with typical spine involvement	716723	1.2	5033	1.1
Other rheumatic diseases without typical spine involvement	1641046	2.7	12366	2.8
Depression	12365147	20.3	5979	19.4
Anxiety disorder	3739348	6.1	19714	4.4
Psychosomatic disorders	7623733	12.5	47025	10.6
Dementia	2148846	3.5	8402	1.9
Sleep disorders	5877886	9.6	6854	10.5
**Physician consultations**				
General practitioner without orthopedic specialist or neurosurgeons	26987138	44.3	110763	24.9
One orthopedic specialist without general practitioner or neurosurgeon	7390065	12.1	29619	6.7
General practitioner and one orthopedic specialist involved	16554747	27.2	127586	28.7
One neurosurgeon without involvement of orthopedic specialist	870269	1.4	26929	6.1
Several orthopedic specialists, neurosurgeons involved	3705824	6.1	128948	29.0
No involvement of general practitioner, orthopedic specialist, neurosurgeon	5434712	8.9	20373	4.6
**Imaging diagnostics**				
Imaging of the spine all forms	25679590	42.1	279917	63.0
Magnetic resonance imaging (MRI)	8270630	13.6	222692	50.1
Computed tomography (CT)	3653329	6.0	94698	21.3
X-ray	19950907	32.7	234455	52.8
Myelography/Neurography	543036	0.9	15626	3.5
**Pain medication**				
NSAIDS	13568544	22.3	357586	80.5
Cox-2 inhibitors	3551595	5.8	53101	12.0
Non-opioid analgesics	16518501	27.1	228983	51.5
Weak opioids	8442994	13.9	156168	35.2
Strong opioids	2583520	4.2	57074	12.8
**Physical therapy**				
Physical therapy all forms	24840083	40.8	229660	51.7
Exercise therapy	14692628	24.1	164542	37.0
Manual therapy	6540786	10.7	61110	13.8
Massage therapy	8046343	13.2	62340	14.0
**Pain therapy**				
Pain therapy care	1171975	1.9	25109	5.7
Spinal manipulative therapy	18716073	30.7	175209	39.4
Acupuncture	4822000	7.9	66709	15.0
Multimodal pain therapy	190310	0.3	7404	1.7
Injection therapy	6025464	9.9	136714	30.8

For episodes with surgery the table entries refer to the two-year period prior to hospitalization when surgery occurred (2008 to 2016)

### Modelling

**Fig. 2:**
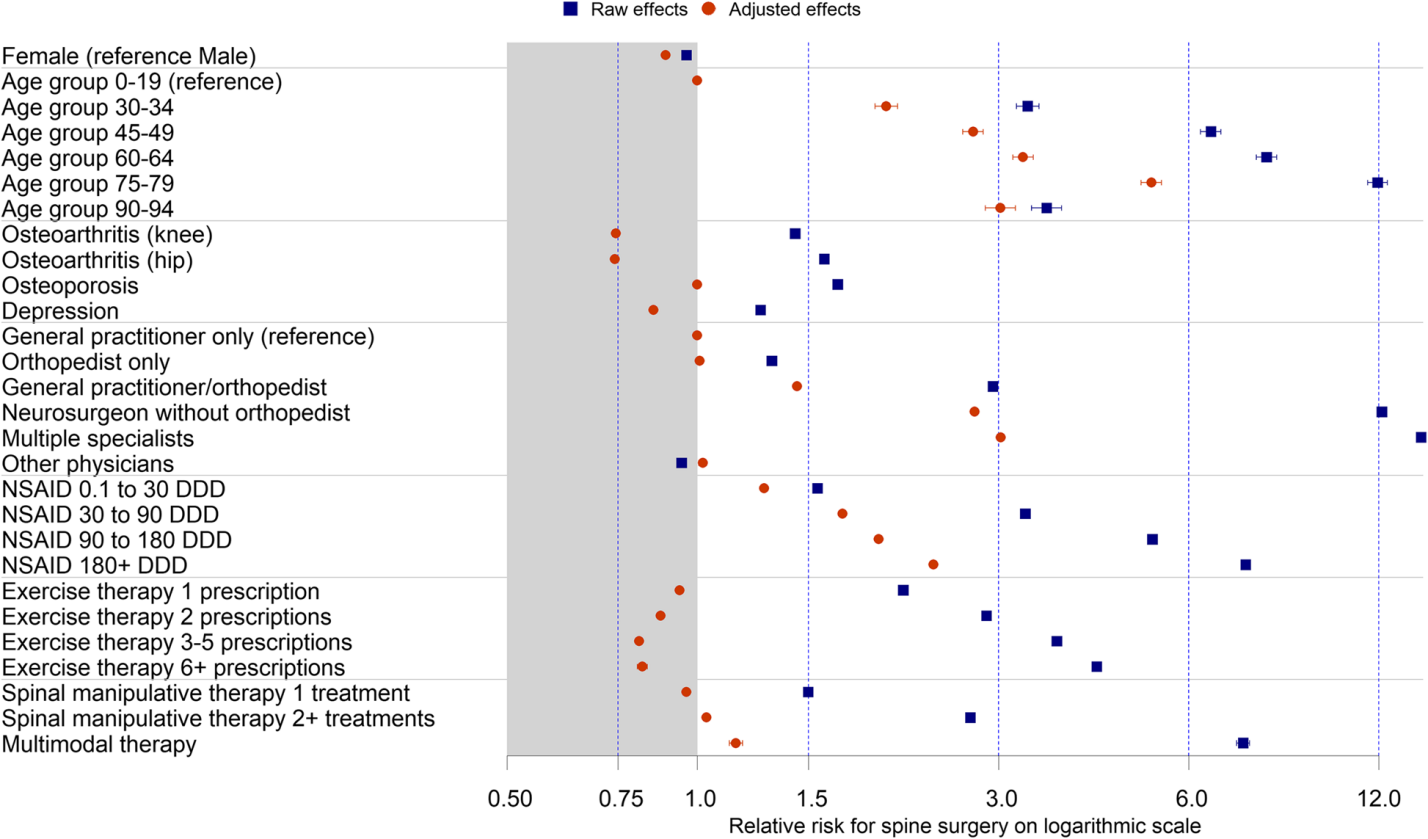
Forest plot for raw and adjusted effect estimates with 95% confidence interval of Poisson regression for selected characteristics for spine interventions based on 60,942,755 two-year episodes of AOK members from 2008 to 2016

**Fig. 3:**
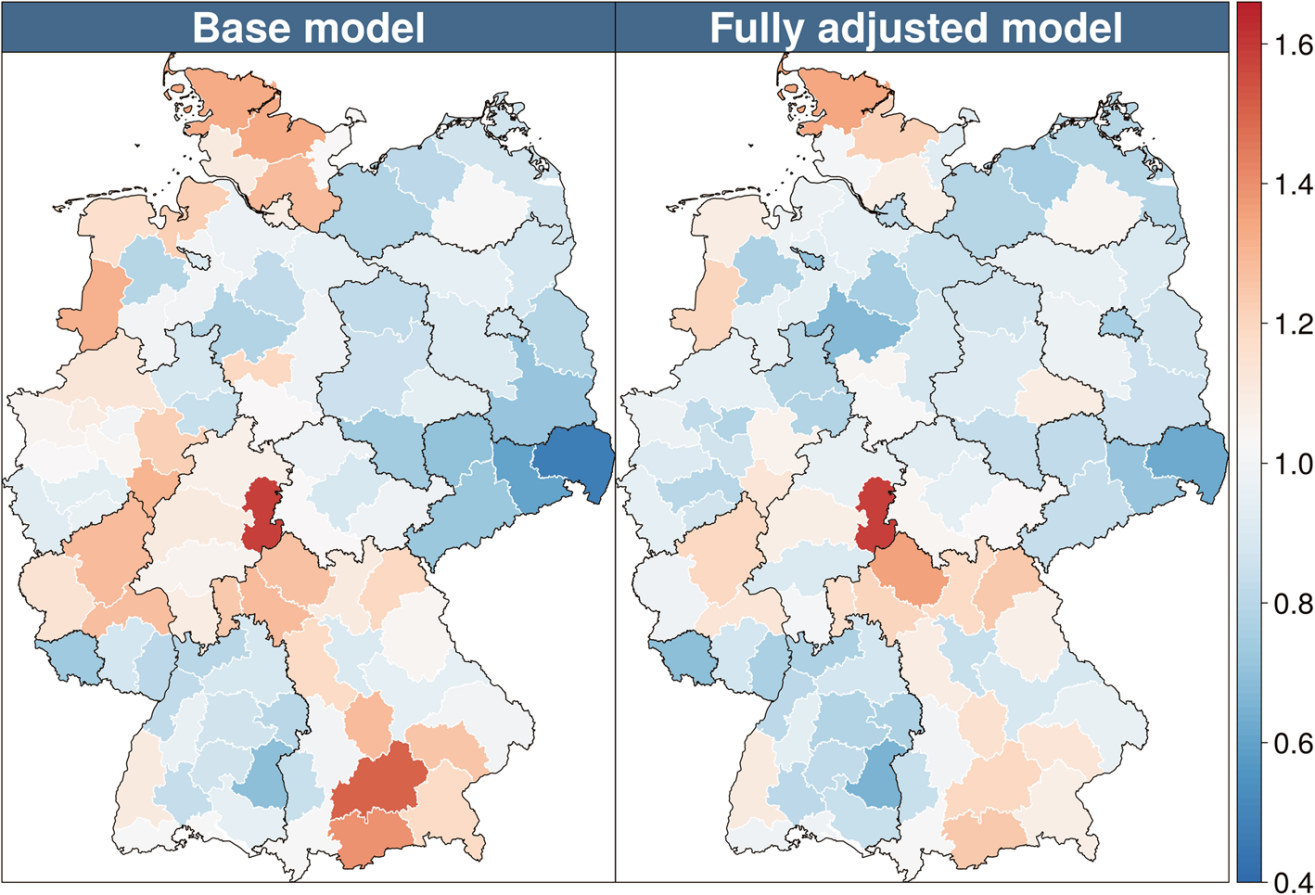
Regional effect estimates for spinal surgery with ICD-10 diagnoses: M40-M54. Shown are relative risks (RR) on a blue (below 1)/red (above 1) spectrum. For the "base model" (left), only age groups and years were controlled for, while the “fully adjusted model” (right) controlled for all variables from Additional file 5 (columns 2–4). Drawn are the borders of the 16 federal states and the areas of the 96 spatial planning regions of Germany. The reference spatial region in the model was the Bavarian region of “Augsburg”. The map is based on data of 60,942,755 patient episodes of the statutory health insurance AOK from 2008 to 2016. To better illustrate the regional differences between the models, the highest estimate (region “Easter Hesse”) was lowered from 1.88 to 1.58 in the left map of the figure

The effect estimates of the Poisson model at the patient episode level without adjustment (raw estimates) and with adjustment for all variables considered are shown for selected variables in the forest plot (Fig. 2) and for regional estimates in Fig. 3. The effect estimates for all model variables are presented in Additional file 5.

### Demographics and morbidity

Females accounted for 56% of the two-year patient episodes with spinal surgery. Compared to males, females had a slightly decreased probability of surgery. With age, the probability of intervention increased up to the 75–79 age group and then decreased in the older age groups (Table 1, Fig. 2). With regard to comorbidities, chronic rheumatoid polyarthritis and osteoporosis were not associated with a higher probability of spinal surgery. Other musculoskeletal conditions such as osteoarthritis of the knee or hip, other rheumatic diseases with and without typical spinal involvement, and psychosomatic disorders, depression, anxiety disorders, sleep disorders, and dementia were associated with a lower probability of surgery in the adjusted model (Additional file 5).

### Physician consultations and imaging diagnostics

The most common treatment pathway for patients with spinal diseases was via the general practitioner alone with 44%. Among all patient episodes with spinal surgery, a quarter of patients were seen only by a general practitioner without involvement of outpatient orthopedic specialist or neurosurgeons. In 29% of the surgery episodes prior contact to two or more orthopedic specialists or neurosurgeons had occurred. Neurosurgeons alone were rarely involved in the care of patients with spinal diseases (1.4%), but for spinal surgery episodes, they were nearly as common (6.1%) as orthopedic specialist alone (6.7%). In 8.9% of the two-year patient episodes without surgery and 4.6% of the patient episodes with surgery no treatment for spinal diseases occurred by any of the three physician groups considered. Magnetic resonance imaging (MRI) was performed in 50%, radiography in 53%, and computed tomography (CT) in 21% of all patients prior to hospitalization for spinal surgery (Table 1).

### Conservative treatments

High-dose Nonsteroidal anti-inflammatory drugs (NSAIDs) was received by 21.8 and 80.5% of patient episodes with spinal diseases/ spinal surgery over a two-year period. As the number of daily doses of NSAIDs received increased, the probability of surgery also increased. Those with spinal surgery had attended physical therapy with indication for the spine in half and spinal manipulation therapy in almost 40% of all episodes (Table 1). More exercise therapy, manual therapy, or massage was associated with a lower probability of surgery (Fig. 2, Additional file 5).

### Regional heterogeneity

The effect of regional entities on spinal surgery is illustrated in Fig. 3. The base model represent the regional effects while controlling for the age distribution in the 96 spatial planning regions within the 16 federal states of Germany. The median region regarding age-standardized spinal surgery rates (Augsburg in the south of Germany) was set as the reference category. The range of the regional estimates of the probability of surgery decreased in the Poisson model from a relative risk of 0.48 to 1.88 (base model) to 0.63 to 1.58 in the fully adjusted model (Fig. 3). Regional clustering as measured by Morans I decreased from the base model to the fully adjusted model from 0.384 to 0.293. By accounting for patient morbidity, physician consultations, imaging diagnostic, pharmacologic as well as non-pharmacological treatments the regional heterogeneity decreased by approximately 25%.

### Sensitivity analyses

In an analysis of retirees only (age > 64), a higher level of needed care was associated with a lower probability of spinal surgery. Inpatient rehabilitation for spine diseases lowered also the probability of surgery in this group. Multimodal pain therapy was associated with a higher probability of surgery for retirees but lower probabilities for the employed group (age 20–64). In the employed group, the probability of surgery was lower for those without a high school diploma or vocational training. Also, the probability of surgery was higher for patients in higher occupational positions. Among employees, those in "business organization, accounting, law, and administration" had the highest probability of spinal surgery. Furthermore, the number of sick leave days for spine diseases in the pre-operative period was strongly associated with surgery (Additional file 5).

## Discussion

This study represents the most comprehensive investigation of spinal surgery in Germany to date. After a sharp increase in surgeries in the past, the annual surgery rate had leveled off at around 300 interventions per 100,000 beneficiaries between 2011 and 2016. Also the regional heterogeneity in surgical care for patients with spine diseases increased during the observation period. The regional differences could only be partially explained by differences in sociodemographic factors, morbidity, consulted physicians, type of imaging diagnostics and use of conservative treatments. After controlling for these factors, the estimates for spinal surgeries in some regions of Germany are twice as high as in other regions. This suggests that other factors, not observable in routine health care data, contribute significantly to the regional heterogeneity of spinal diseases. This also underlines the necessity to formulate and follow evidence-based disease specific indication criteria for spinal surgery.

In this regard the study allows numerous takeaways about patterns of care and associated factors influencing the indication for spinal surgery. For example, the study shows that one in four patients who underwent spinal surgery consulted solely a general practitioner in the two-year period prior to surgery and were not treated by a practicing orthopedic specialist or neurosurgeon. This concerns more than 110,000 surgical cases in the study period. Magnetic resonance imaging was performed in only 50% and computed tomography in 29% of spine surgery patients before hospital admission. This fact raises the question if the indication for spinal surgery and transferal to a specialized hospital can be done without MRI or CT or if a high proportion of the patients had an acute onset, which demanded an emergency hospitalization. The access to medical care within the spatial region was not measured in this study. The effect of consulting a specialist was controlled, but not the likelihood of consulting one. The heterogeneity of medical care within a region may have resulted in some patients migrating to the inpatients sector.

Four-fifths of the patients who underwent spinal surgery received NSAIDs pain medication at the expense of the insurance prior to surgery. Also only half of the patients received physical therapy with indication for the spine in the two years before the surgery. The utilization of physical therapy and pain medications before surgery was similar to the results reported for hip and knee replacement in Germany [16, 17].

Remarkably, after controlling for all other variables’ representative of disease severity, patients with physical therapy were less likely to undergo surgery than patients without. This effect was larger for higher amounts of physical therapy prescriptions. The even lower probability of surgery after conservative treatments in the subgroup of employees could be explained by the inclusion of additional variables (i.e., days of sick leave, social status). The differences in intervention probabilities depending on spinal manipulation therapy and multimodal pain therapy should be interpreted cautiously. The weights used to approximate the severity of the spinal disease may have differed between the study groups, which could have led to an underestimation of the effect estimate of these factors in the regression model.

The Atlas of Care for Musculoskeletal Conditions used aggregated Medicare data in the USA to investigate regional differences in surgeries 20 years ago. It was shown that the regional variation in surgeries was lowest for hip fractures and highest for spinal procedures [5]. This variation was explained by different treatment preferences among the treating physicians. These preferences could be based either on insufficient scientific evidence for the treatment or on different benefit-risk ratios of the treatment options. The variation of this ratio could be due to different integration of patient preferences [5]. Our analysis supports the idea that even after controlling for numerous individual factors, physicians’ preferences between and within the same medical specialty might play a major role regarding the choice of care for spinal diseases.

Only one other smaller cohort study with data from the Washington State Department of Labor and Industries State Fund can be used for comparison with the present study. In this study 9.2% of the employees received spinal surgery in the three years following an occupational back injury. In addition to the characteristics of disease severity (i.e., clinical status, Roland Morris Disability Score, pain intensity), the first physician consulted after the accident was essential. Compared to the general practitioner, patients who had first seen a surgeon had a much higher and those who had first seen a chiropractor had a lower probability for surgery [18].

### Strengths and limitations

The main strengths of the study are the long observation period, the large number of patients, and the inclusion of a large number of relevant variables associated with the probability of surgery. The main limitation of the study is the fact that the data were collected for documentation and billing purposes, leading to missing information on specific symptoms and the inability to detect undiagnosed diseases due to low health seeking behavior. Furthermore, there are potential limitations due to patients with spinal diseases often receiving several ICD-10 codes in the M40-M54 group in the outpatient setting. This makes it difficult to accurately record the clinical disease and define the population at risk for certain surgeries. But it reflects the fact that the majority of patients with low back pain can often not get a precise pathoanatomical diagnosis [19]. Outpatient rehabilitation/rehabilitation sports and inpatient rehabilitation for employees could due to limited data availability not be included in the analysis. Services that are not billed to the health insurance fund are also not recorded and therefore not included in the analysis, which is the case for low dose NASID medications. Based on survey data, the prevalence for chronic back pain is higher for beneficiaries in the AOK than in other health insurances in Germany [20]. This reflects differences in membership structure between insurances, but should not differ for the group of patients with spinal diseases within each health insurance, since all patients are treated by the same physicians and hospitals.

In addition, routine healthcare data alone cannot accurately reflect health behavior. Patients who are more active or more health-conscious may be more willing to use physical therapy or demand it from their health care providers. The resulting "healthy user bias" could overestimate the effects for these therapies [21]. Additionally, the fact that the severity of the disease was only approximated could underestimate the effect of these therapies.

## Conclusions

The analyses presented show that monitoring of medical care on the basis of comprehensive routine health care data offers valuable insight into individual as well as regional care patters. These findings call for the development and adherence to evidence-based, uniform indication criteria for spinal surgery. They also should stimulate further research in the evaluation of mandatory pre surgery diagnostic and treatment procedures and structural paths of care for patients with diseases of the lumbar spine.

## Supplementary Information

**Additional file 1. **Definition of spinal surgery.

**Additional file 2. **Definition of comorbidities.

**Additional file 3. **Definition of diagnostics and treatments.

**Additional file 4. **Definition of pain medication.

**Additional file 5. **Regression results.

**Additional file 6. **Extended description of cohort and subcohorts.

## Data Availability

The authors confirm that the data utilized in this study cannot be made available in the manuscript, the supplemental files, or in a public repository due to German data protection laws (‘Bundesdatenschutzgesetz’, BDSG). Therefore, they are stored on a secure drive in the WIdO, to facilitate replication of the results. Generally, access to data of statutory health insurance funds for research purposes is possible only under the conditions defined in German Social Law (SGB V § 287). Requests for data access can be sent as a formal proposal specifying the recipient and purpose of the data transfer to the appropriate data protection agency. Access to the data used in this study can only be provided to external parties under the conditions of the cooperation contract of this research project and after written approval by the sickness fund. For assistance in obtaining access to the data, please contact wido@wido.bv.aok.de. The R code of the analysis can be made available upon request by the corresponding author.

## Abbreviations

AOK: “Allgemeine Ortskrankenkassen” a statutory health insurance in Germany
CI: Confidence interval
CT: Computed tomography
ICD-10: International Statistical Classification of Diseases and Related Health Problems, 10th revision
MRI: Magnetic resonance imaging
NSAID: Nonsteroidal anti-inflammatory drugs
OPS: Official classification code of operations and procedures in Germany
RR: Relative Risk as effect estimates in a Poisson regression model
