# Supplementary material for: Indication for spinal surgery: associated factors and regional differences in Germany
Source: BMC Health Serv Res. 2022 Sep 1;22:1109. doi: 10.1186/s12913-022-08492-3 (PMC9438246; doi:10.1186/s12913-022-08492-3)
Supplement: Supplementary file 1 — Additional file 1. Definition of spinal surgery. [file 12913_2022_8492_MOESM1_ESM.docx]

**Supplementary Material**

Additional file 1: Classification of OPS codes for spinal surgery 2008-2016

| **Spine Surgery group** | **Operation and procedure codes** |
| --- | --- |
| Disc and bone excision | 5831a, 58310, 58311, 58312, 58313, 58314, 58315, 58318, 58319, 5832x, 5832y, 58320, 58321, 58322, 58323, 58324, 58325, 58326, 58327, 58328, 58329 |
| Disc recurrence | 58316, 58317 |
| Fusions (reduction, osteosynthesis, spondylodesis) | 583bx, 583bx0, 583bx1, 583bx2, 583bx3, 583by, 583b0, 583b00, 583b01, 583b02, 583b03, 583b1, 583b10, 583b11, 583b12, 583b13, 583b2, 583b20, 583b21, 583b22, 583b23, 583b3, 583b30, 583b31, 583b32, 583b33, 583b4, 583b40, 583b41, 583b42, 583b43, 583b5, 583b50, 583b51, 583b52, 583b53, 583b6, 583b60, 583b61, 583b62, 583b63, 583b7, 583b70, 583b71, 583b72, 583b73, 583b8, 583w0, 583w1, 5833x, 5833y, 5834x, 5834y, 58340, 58341, 58342, 58343, 58344, 58345, 58346, 5835a0, 5835a1, 5835b0, 5835b1, 5835c, 5835d, 5835e, 5835x, 5835y, 58350, 58351, 58352, 58353, 58354, 58355, 58356, 583580, 583581, 583582, 583583, 58359, 5836x, 5836y, 583630, 583631, 583632, 583633, 583640, 583641, 583642, 583650, 583651, 583653 |
| Vertebral body replacement | 5837a0, 5837a1, 5837a2, 5837a3, 5837x, 5837y, 583700, 583701, 583702, 583704, 583705, 58371, 58372, 58373, 58374, 58375, 58376 |
| Scoliosis | 5838a2, 5838a3, 5838a4, 5838a5, 5838a6, 5838b0, 5838b1, 5838b2, 5838b3, 5838b4, 5838b5, 5838d0, 5838d1, 5838d3, 5838e0, 5838e1, 5838e2, 5838e3, 5838f0, 5838f1, 5838x, 58382, 58383, 583892, 583893, 583894, 583895, 583896, 583897 |
| spinal decompression | 583960, 583961, 583962, 583963 |
| Spreader | 5839b0, 5839b1 |
| spreader removal | 5839c0, 5839c1, 5839d0, 5839d1, 5839f0, 5839f1, 5839f2, 5839g0, 5839g1, 5839g2, 5839h0, 5839h1, 5839h2, 5839h3 |
| Facet surgery | 583a00, 583a01, 583a02, 58302 |
| disc endoprosthesis | 583910, 583911, 583912, 583913 |
| Intervertebral disc endoprosthesis, revision: | 58392, 58393, 58394 |
| Kyphoplasty, implantation material vertebral body | 5839a0, 5839a1, 5839a2, 5839a3, 583990, 583991, 583992, 583993 |
| Minimally invasive treatment procedures on the spine | 583ax, 583ay, 583a0, 583a1, 583a10, 583a11, 583a12 |
| Revision, material ex | 5830x, 5830y, 58303, 58304, 58305, 58306, 58390, 58395 |
