# Supplementary material for: Indication for spinal surgery: associated factors and regional differences in Germany
Source: BMC Health Serv Res. 2022 Sep 1;22:1109. doi: 10.1186/s12913-022-08492-3 (PMC9438246; doi:10.1186/s12913-022-08492-3)
Supplement: Supplementary file 2 — Additional file 2. Definition of comorbidities. [file 12913_2022_8492_MOESM2_ESM.docx]

**Supplementary Material**

Additional file 2: Definition of Comorbidities

| **Diseases** | **ICD-10-GM Codes** | **Definition** |
| --- | --- | --- |
| Fractures of the spine | S12/S22/S32 | Once coded in outpatient or inpatient sector |
| Osteoarthritis (knee) | M16 | M2Q* |
| Osteoarthritis (hip) | M17 | M2Q |
| Osteoporosis | M80-82 | M2Q |
| Chronic rheumatoid polyarthritis | M05, M06, M08, M09 | M2Q |
| Other rheumatic diseases with typical spine involvement | M7, M45, M46.1, M46.8, M46.9 | M2Q |
| Other rheumatic diseases without typical spine involvement | L94.0, L94.1, L94.3, M12.0, M12.3,M30, M31, M32, M33, M34, M35.0-6, M36.0, , | M2Q |
| Depression | F32,F33,F34.1 | M2Q |
| Anxiety disorder | F40,F41 | M2Q |
| Psychosomatic disorders | F45 | M2Q |
| Dementia | G30, G31.0, G31.82, G23.1, F00, F01, F02, F03,F05.1 | M2Q |
| Sleep disorders | G47,G25.8,F51 | M2Q |

*M2Q (At least coded in two quarters within 4 quarters with secured diagnostic certainty)
