# Supplementary material for: Indication for spinal surgery: associated factors and regional differences in Germany
Source: BMC Health Serv Res. 2022 Sep 1;22:1109. doi: 10.1186/s12913-022-08492-3 (PMC9438246; doi:10.1186/s12913-022-08492-3)
Supplement: Supplementary file 3 — Additional file 3. Definition of diagnostics and treatments. [file 12913_2022_8492_MOESM3_ESM.docx]

**Supplementary Material**

Additional file 3: Treatment definitions

| **Treatment** | **German Billing Code** |
| --- | --- |
|  | **Remedy position** |
| Massage therapy | X0101 – X0108 with Indication WS (Spine) |
| Exercise therapy (1 to 1) | X0301 – X0308, X0501 – X0506 with Indication WS (Spine) |
| Manual therapy | X1201 – X1204 with Indication WS (Spine) |
|  | **Outpatient code (EBM)** |
| Pain therapy care | 30702 (ab 2008) |
| Acupuncture | 30791 (ab 2007) |
| Spinal manipulation therapy | 30201 |
| Outpatient Injection therapy | 30722, 30723, 30724, 30731, 02360, 34503 |
| MRI | 34411 |
| CT | 34311 |
| X-ray | 34221, 34222 |
| Myelography/Neurography | 34223 |
|  | **Operation and procedure code (OPS)** |
| Inpatient Injection therapy | 8-910,8-911,8-913,8-914,8-915,8-916,8-917, ,8-919 |
| Multimodal pain therapy | 8-918,8-91b, 8-91c (ab 2009) |
| MRI | 3-802, 3-823 |
| CT | 3-203, 3-223 |
| Myelographie/Neurographie | 3-841,3-130,1-206 |
