# Supplementary material for: Indication for spinal surgery: associated factors and regional differences in Germany
Source: BMC Health Serv Res. 2022 Sep 1;22:1109. doi: 10.1186/s12913-022-08492-3 (PMC9438246; doi:10.1186/s12913-022-08492-3)
Supplement: Supplementary file 4 — Additional file 4. Definition of pain medication. [file 12913_2022_8492_MOESM4_ESM.docx]

**Supplementary Material**

Additional file 4: Pain medication

| **Group** | **ATC-Code** | **Active ingredient** |
| --- | --- | --- |
| NSAID | M01AA | Butylpyrazolidines |
|  | M01AB | Acetic acid derivatives and related substances |
|  | M01AC | Oxicams |
|  | M01AE | Propionic acid derivatives |
|  | N02BA | Salicylic acid and derivatives |
| Cox-2 inhibitors | M01AH | Coxibe |
| Non-opioid analgesics | N02BB | Pyrazolones |
|  | N02BE | Anilides |
|  | N02BG | Other analgesics and antipyretics |
| weak-acting opioids | N02AA08 | Dihydrocodeine |
|  | N02AA57 | Ethyl morphine, combinations |
|  | N02AA58 | Dihydrocodeine, combinations |
|  | N02AA59 | Codeine, comb. excl. psycholeptics |
|  | N02AA65 | Codeine and diclofenac |
|  | N02AA66 | Codeine and acetylsalicylic acid |
|  | N02AA69 | Codeine and paracetamol |
|  | N02AA79 | Codeine, combinations with psycholeptics |
|  | N02AX01 | Tilidine |
|  | N02AX02 | Tramadol |
|  | N02AX05 | Meptazinol |
|  | N02AX51 | Tilidine and naloxone |
|  | N02AX53 | Tramadol and dexketoprofen |
|  | N02AX62 | Tramadol and paracetamol |
| strong-acting opioids | N01AH03 | Sufentanil |
|  | N02AA01 | Morphine |
|  | N02AA02 | Opium |
|  | N02AA03 | Hydromorphone |
|  | N02AA04 | Nicomorphine |
|  | N02AA05 | Oxycodone |
|  | N02AA51 | Morphine, combinations |
|  | N02AA55 | Oxycodone combinations |
|  | N02AB02 | Pethidine |
|  | N02AB03 | Fentanyl |
|  | N02AB52 | Pethidine, comb. excl. psycholeptics |
|  | N02AB72 | Pethidine, combinations with psycholeptics |
|  | N02AC03 | Piritramide |
|  | N02AC06 | Levomethadone |
|  | N02AC52 | Methadone, comb. excl. psycholeptics |
|  | N02AD01 | Pentazocine |
|  | N02AD02 | Phenazocine |
|  | N02AE01 | Buprenorphine |
|  | N02AF02 | Nalbufin |
|  | N02AG01 | Morphine with spasmolytics |
|  | N02AG02 | Ketobemidone with spasmolytics |
|  | N02AG03 | Pethidine with spasmolytics |
|  | N02AG04 | Hydromorphone with spasmolytics |
|  | N02AX03 | Dezocin |
|  | N02AX06 | Tapentadol |
