# Supplementary material for: Indication for spinal surgery: associated factors and regional differences in Germany
Source: BMC Health Serv Res. 2022 Sep 1;22:1109. doi: 10.1186/s12913-022-08492-3 (PMC9438246; doi:10.1186/s12913-022-08492-3)
Supplement: Supplementary file 5 — Additional file 5. Regression results. [file 12913_2022_8492_MOESM5_ESM.docx]

**Supplementary Material**

Additional file 5: Poisson regression analysis: relative risks (RR) for spinal surgery from 2008 to 2016 are shown at the patient level, for employees only from 2012 to 2016. Effects of individual years and 96 spatial regions are not shown. Patients with fractures of the spine were excluded.

|  | **All 2008-16** | | | **Employed 2012-16** | | | **Retired 2008-16** | | |
| --- | --- | --- | --- | --- | --- | --- | --- | --- | --- |
|  | **RR** | **95 % CI** | | **RR** | **95 % CI** | | **RR** | **95 % CI** | |
| **Demographics** |  |  | |  |  | |  |  | |
| Female (Ref. Male) | 0.892 | 0.886 | 0.897 | 1.105 | 1.080 | 1.130 | 0.958 | 0.949 | 0,966 |
| Age group 0-19 | 1(Ref.) |  |  |  |  |  |  |  |  |
| Age group 20-24 | 0.885 | 0.838 | 0.935 | 1(Ref.) |  |  |  |  |  |
| Age group 25-29 | 1.464 | 1.400 | 1.532 | 1.443 | 1.305 | 1.596 |  |  |  |
| Age group 30-34 | 1.993 | 1.913 | 2.077 | 1.733 | 1.575 | 1.907 |  |  |  |
| Age group 35-39 | 2.317 | 2.228 | 2.410 | 1.820 | 1.658 | 1.998 |  |  |  |
| Age group 40-44 | 2.624 | 2.527 | 2.726 | 2.006 | 1.832 | 2.197 |  |  |  |
| Age group 45-49 | 2.734 | 2.634 | 2.838 | 2.038 | 1.863 | 2.229 |  |  |  |
| Age group 50-54 | 2.877 | 2.772 | 2.986 | 2.121 | 1.940 | 2.320 |  |  |  |
| Age group 55-59 | 3.061 | 2.950 | 3.177 | 2.100 | 1.919 | 2.296 |  |  |  |
| Age group 60-64 | 3.281 | 3.161 | 3.405 | 1.744 | 1.591 | 1.911 |  |  |  |
| Age group 65-69 | 4.299 | 4.143 | 4.461 |  |  |  | 1(Ref.) |  |  |
| Age group 70-74 | 4.971 | 4.792 | 5.157 |  |  |  | 1.151 | 1.137 | 1,164 |
| Age group 75-79 | 5.237 | 5.047 | 5.434 |  |  |  | 1.199 | 1.185 | 1,214 |
| Age group 80-84 | 4.952 | 4.769 | 5.142 |  |  |  | 1.136 | 1.120 | 1,153 |
| Age group 85-89 | 4.239 | 4.072 | 4.413 |  |  |  | 0.992 | 0.971 | 1,012 |
| Age group 90-94 | 3.021 | 2.858 | 3.193 |  |  |  | 0.741 | 0.709 | 0,774 |
| Age group 95+ | 1.506 | 1.312 | 1.729 |  |  |  | 0.421 | 0.368 | 0,481 |
| **Comorbidities** |  |  |  |  |  |  |  |  |  |
| Osteoarthritis (knee) | 0.744 | 0.737 | 0.751 | 0.641 | 0.614 | 0.670 | 0.765 | 0.757 | 0,773 |
| Osteoarthritis (hip) | 0.741 | 0.735 | 0.747 | 0.723 | 0.699 | 0.749 | 0.761 | 0.754 | 0,768 |
| Osteoporosis | 1.000 | 0.990 | 1.010 | 0.814 | 0.759 | 0.873 | 1.008 | 0.998 | 1,019 |
| Chronic rheumatoid polyarthritis | 1.088 | 1.072 | 1.104 | 1.024 | 0.962 | 1.090 | 1.126 | 1.106 | 1,146 |
| Other rheumatic diseases with typical spine involvement | 0.757 | 0.736 | 0.779 | 0.565 | 0.518 | 0.616 | 0.874 | 0.841 | 0,909 |
| Other rheumatic diseases without typical spine involvement | 0.816 | 0.802 | 0.831 | 0.737 | 0.662 | 0.821 | 0.841 | 0.824 | 0,858 |
| Depression | 0.853 | 0.846 | 0.860 | 0.693 | 0.674 | 0.712 | 0.897 | 0.887 | 0,906 |
| Anxiety disorder | 0.841 | 0.829 | 0.853 | 0.788 | 0.752 | 0.827 | 0.878 | 0.860 | 0,897 |
| Psychosomatic disorders | 0.650 | 0.643 | 0.657 | 0.550 | 0.532 | 0.569 | 0.699 | 0.690 | 0,709 |
| Dementia | 0.910 | 0.901 | 0.919 | 0.874 | 0.842 | 0.906 | 0.940 | 0.929 | 0,952 |
| Sleep disorders | 0.705 | 0.690 | 0.721 |  |  |  | 0.786 | 0.768 | 0,805 |
| **Physician consultations** |  |  |  |  |  |  |  |  |  |
| General practitioner | 1(Ref.) |  |  | 1(Ref.) |  |  | 1(Ref.) |  |  |
| One orthopedic specialist | 1.440 | 1.427 | 1.454 | 0.913 | 0.888 | 0.939 | 1.381 | 1.363 | 1,399 |
| General practitioner and one orthopedic specialist | 1.009 | 0.996 | 1.023 | 1.041 | 0.995 | 1.089 | 1.042 | 1.024 | 1,061 |
| One neurosurgeon without involvement of orthopedic specialist | 2.749 | 2.710 | 2.789 | 1.414 | 1.361 | 1.468 | 2.457 | 2.404 | 2,510 |
| Several orthopedic specialists /neurosurgeons | 1.021 | 1.005 | 1.036 | 1.352 | 1.288 | 1.419 | 1.080 | 1.059 | 1,102 |
| No involvement of general practitioner, orthopedic specialist, neurosurgeon | 3.026 | 2.993 | 3.060 | 1.701 | 1.650 | 1.754 | 2.805 | 2.762 | 2,849 |
| **Imaging** **of the spine** |  |  |  |  |  |  |  |  |  |
| MRI none | 1(Ref.) |  |  | 1(Ref.) |  |  | 1(Ref.) |  |  |
| MRI 1 | 3.393 | 3.367 | 3.419 | 1.459 | 1.427 | 1.492 | 3.134 | 3.101 | 3,168 |
| MRI 2+ | 3.889 | 3.847 | 3.932 | 1.588 | 1.540 | 1.636 | 3.206 | 3.153 | 3,259 |
| CT none | 1(Ref.) |  |  | 1(Ref.) |  |  | 1(Ref.) |  |  |
| CT 1 | 1.852 | 1.837 | 1.868 | 1.072 | 1.044 | 1.101 | 1.823 | 1.802 | 1,844 |
| CT 2+ | 1.236 | 1.215 | 1.258 | 0.700 | 0.657 | 0.745 | 1.204 | 1.175 | 1,233 |
| X-ray none | 1(Ref.) |  |  | 1(Ref.) |  |  | 1(Ref.) |  |  |
| X-ray 1 | 1.327 | 1.318 | 1.337 | 1.084 | 1.062 | 1.106 | 1.368 | 1.354 | 1,382 |
| X-ray 2+ | 1.353 | 1.340 | 1.367 | 1.037 | 1.007 | 1.068 | 1.429 | 1.409 | 1,449 |
| Myelography/Electroneurography none | 1(Ref.) |  |  | 1(Ref.) |  |  | 1(Ref.) |  |  |
| Myelography/ Electroneurography 1 | 1.343 | 1.321 | 1.367 | 1.082 | 1.019 | 1.148 | 1.380 | 1.349 | 1,413 |
| Myelography/ Electroneurography 2+ | 0.279 | 0.263 | 0.295 | 0.163 | 0.128 | 0.208 | 0.309 | 0.287 | 0,333 |
| **Pain medication** |  |  |  |  |  |  |  |  |  |
| NSAID none | 1(Ref.) |  |  | 1(Ref.) |  |  | 1(Ref.) |  |  |
| NSAID 0.1 to 30 DDD | 1.276 | 1.263 | 1.288 | 1.183 | 1.149 | 1.218 | 1.235 | 1.217 | 1,252 |
| NSAID 30 to 90 DDD | 1.698 | 1.682 | 1.713 | 1.279 | 1.244 | 1.314 | 1.478 | 1.459 | 1,497 |
| NSAID 90 to 180 DDD | 1.939 | 1.919 | 1.960 | 1.386 | 1.342 | 1.431 | 1.679 | 1.655 | 1,703 |
| NSAID 180+ DDD | 2.367 | 2.344 | 2.391 | 1.764 | 1.706 | 1.824 | 2.161 | 2.133 | 2,189 |
| Cox-2 inhibitors none | 1(Ref.) |  |  | 1(Ref.) |  |  | 1(Ref.) |  |  |
| Cox-2 inhibitors 0.1 to 30 DDD | 0.863 | 0.849 | 0.877 | 0.698 | 0.665 | 0.734 | 0.859 | 0.840 | 0,879 |
| Cox-2 inhibitors 30 to 90 DDD | 1.788 | 1.763 | 1.815 | 1.743 | 1.675 | 1.813 | 1.744 | 1.709 | 1,779 |
| Cox-2 inhibitors 90 to 180 DDD | 1.205 | 1.177 | 1.233 | 1.117 | 1.040 | 1.199 | 1.201 | 1.164 | 1,239 |
| Cox-2 inhibitors 180+ DDD | 1.573 | 1.542 | 1.604 | 1.594 | 1.489 | 1.706 | 1.565 | 1.525 | 1,606 |
| Non-opioid analgesics none | 1(Ref.) |  |  | 1(Ref.) |  |  | 1(Ref.) |  |  |
| Non-opioid analgesics 0.1 to 30 DDD | 1.391 | 1.382 | 1.401 | 1.123 | 1.101 | 1.145 | 1.413 | 1.399 | 1,427 |
| Non-opioid analgesics 30 to 90 DDD | 1.459 | 1.443 | 1.475 | 1.134 | 1.094 | 1.175 | 1.514 | 1.492 | 1,535 |
| Non-opioid analgesics 90 to 180 DDD | 1.419 | 1.394 | 1.444 | 1.161 | 1.077 | 1.251 | 1.486 | 1.453 | 1,518 |
| Non-opioid analgesics 180+ DDD | 1.590 | 1.558 | 1.622 | 1.241 | 1.118 | 1.376 | 1.710 | 1.668 | 1,753 |
| weak-acting opioids none | 1(Ref.) |  |  | 1(Ref.) |  |  | 1(Ref.) |  |  |
| weak-acting opioids 0.1 to 30 DDD | 1.462 | 1.450 | 1.475 | 1.074 | 1.048 | 1.100 | 1.404 | 1.387 | 1,420 |
| weak-acting opioids 30 to 90 DDD | 1.430 | 1.414 | 1.447 | 1.065 | 1.026 | 1.106 | 1.372 | 1.351 | 1,393 |
| weak-acting opioids 90 to 180 DDD | 1.302 | 1.279 | 1.325 | 1.012 | 0.943 | 1.086 | 1.275 | 1.246 | 1,304 |
| weak-acting opioids 180+ DDD | 1.568 | 1.547 | 1.589 | 1.313 | 1.238 | 1.393 | 1.547 | 1.521 | 1,573 |
| strong-acting opioids none | 1(Ref.) |  |  | 1(Ref.) |  |  | 1(Ref.) |  |  |
| strong-acting opioids 0.1 to 30 DDD | 1.176 | 1.160 | 1.191 | 0.936 | 0.892 | 0.982 | 1.228 | 1.207 | 1,248 |
| strong-acting opioids 30 to 90 DDD | 1.104 | 1.081 | 1.127 | 0.982 | 0.893 | 1.079 | 1.170 | 1.140 | 1,200 |
| strong-acting opioids 90 to 180 DDD | 1.061 | 1.033 | 1.091 | 1.022 | 0.891 | 1.172 | 1.133 | 1.096 | 1,171 |
| strong-acting opioids 180+ DDD | 1.517 | 1.492 | 1.543 | 1.351 | 1.225 | 1.490 | 1.551 | 1.518 | 1,585 |
| **Pain therapy** |  |  |  |  |  |  |  |  |  |
| Pain therapy care none | 1(Ref.) |  |  | 1(Ref.) |  |  | 1(Ref.) |  |  |
| Pain therapy care 1 | 1.154 | 1.131 | 1.177 | 1.138 | 1.075 | 1.204 | 1.122 | 1.090 | 1,155 |
| Pain therapy care 2+ | 1.193 | 1.172 | 1.215 | 1.346 | 1.271 | 1.426 | 1.164 | 1.136 | 1,193 |
| Spinal manipulative therapy none | 1(Ref.) |  |  | 1(Ref.) |  |  | 1(Ref.) |  |  |
| Spinal manipulative therapy | 0.962 | 0.954 | 0.971 | 0.999 | 0.975 | 1.025 | 0.984 | 0.971 | 0,997 |
| Spinal manipulative therapy | 1.035 | 1.027 | 1.044 | 1.040 | 1.016 | 1.065 | 1.046 | 1.034 | 1,058 |
| Acupuncture none | 1(Ref.) |  |  | 1(Ref.) |  |  | 1(Ref.) |  |  |
| Acupuncture till 10 sessions | 1.100 | 1.089 | 1.111 | 0.991 | 0.961 | 1.021 | 1.112 | 1.097 | 1,127 |
| Acupuncture 11+ sessions | 1.236 | 1.217 | 1.255 | 1.001 | 0.948 | 1.058 | 1.280 | 1.256 | 1,306 |
| Multimodal pain therapy | 1.152 | 1.125 | 1.180 | 0.893 | 0.831 | 0.959 | 1.102 | 1.064 | 1,141 |
| Injection therapy none | 1(Ref.) |  |  | 1(Ref.) |  |  | 1(Ref.) |  |  |
| Injection therapy 1 | 1.311 | 1.298 | 1.325 | 1.078 | 1.046 | 1.110 | 1.239 | 1.221 | 1,257 |
| Injection therapy 2+ | 1.525 | 1.512 | 1.538 | 1.262 | 1.231 | 1.294 | 1.412 | 1.395 | 1,429 |
| **Physical Therapy (Indication Spine)** |  |  |  |  |  |  |  |  |  |
| Exercise therapy none | 1(Ref.) |  |  | 1(Ref.) |  |  | 1(Ref.) |  |  |
| Exercise therapy 1 Prescription | 0.938 | 0.930 | 0.946 | 0.759 | 0.741 | 0.777 | 0.964 | 0.952 | 0,975 |
| Exercise therapy 2 Prescription | 0.875 | 0.865 | 0.884 | 0.640 | 0.621 | 0.659 | 0.898 | 0.884 | 0,912 |
| Exercise therapy 3-5 Prescription | 0.810 | 0.801 | 0.820 | 0.546 | 0.530 | 0.563 | 0.842 | 0.829 | 0,856 |
| Exercise therapy 6+ Prescription | 0.819 | 0.804 | 0.834 | 0.526 | 0.497 | 0.556 | 0.849 | 0.828 | 0,870 |
| Manual therapy none | 1(Ref.) |  |  | 1(Ref.) |  |  | 1(Ref.) |  |  |
| Manual therapy 1 Prescription | 0.961 | 0.949 | 0.972 | 0.963 | 0.934 | 0.994 | 0.958 | 0.942 | 0,975 |
| Manual therapy 2 Prescription | 0.937 | 0.921 | 0.953 | 0.843 | 0.806 | 0.881 | 0.945 | 0.922 | 0,968 |
| Manual therapy 3-5 Prescription | 0.941 | 0.924 | 0.959 | 0.791 | 0.754 | 0.830 | 0.970 | 0.945 | 0,995 |
| Manual therapy 6+ Prescription | 1.002 | 0.968 | 1.037 | 0.847 | 0.772 | 0.928 | 1.022 | 0.974 | 1,071 |
| Massage therapy none | 1(Ref.) |  |  | 1(Ref.) |  |  | 1(Ref.) |  |  |
| Massage therapy 1 Prescription | 0.889 | 0.880 | 0.898 | 0.849 | 0.821 | 0.877 | 0.923 | 0.910 | 0,937 |
| Massage therapy 2 Prescription | 0.868 | 0.853 | 0.883 | 0.785 | 0.740 | 0.833 | 0.908 | 0.888 | 0,929 |
| Massage therapy 3-5 Prescription | 0.859 | 0.841 | 0.878 | 0.852 | 0.786 | 0.923 | 0.900 | 0.874 | 0,926 |
| Massage therapy 6+ Prescription | 0.931 | 0.886 | 0.979 | 0.870 | 0.674 | 1.124 | 0.963 | 0.901 | 1,029 |
| **Rehabilitation/Needed care** |  |  |  |  |  |  |  |  |  |
| inpatient rehabilitation |  |  |  |  |  |  | 0.925 | 0.878 | 0,976 |
| Needed care none |  |  |  |  |  |  | 1(Ref.) |  |  |
| Needed care stage I |  |  |  |  |  |  | 0.793 | 0.779 | 0,807 |
| Needed care stage II |  |  |  |  |  |  | 0.533 | 0.516 | 0,550 |
| Needed care stage III |  |  |  |  |  |  | 0.190 | 0.166 | 0,217 |
| **Sick leave days** |  |  |  |  |  |  |  |  |  |
| none |  |  |  | 1(Ref.) |  |  |  |  |  |
| 1-7 days |  |  |  | 1.488 | 1.382 | 1.603 |  |  |  |
| 8-21 days |  |  |  | 2.860 | 2.684 | 3.048 |  |  |  |
| 22-42 days |  |  |  | 8.758 | 8.265 | 9.280 |  |  |  |
| 42+ days |  |  |  | 117.1 | 112.0 | 122.4 |  |  |  |
| **Education** |  |  |  |  |  |  |  |  |  |
| Unknown school-leaving qualification |  |  |  | 0.958 | 0.909 | 1.010 |  |  |  |
| No school-leaving qualification |  |  |  | 0.844 | 0.781 | 0.911 |  |  |  |
| Lower Secondary leaving certificate |  |  |  | 1.011 | 0.960 | 1.064 |  |  |  |
| Intermediate school leaving certificate |  |  |  | 1.001 | 0.951 | 1.055 |  |  |  |
| High school diploma |  |  |  | 1(Ref.) |  |  |  |  |  |
| Unknown vocational training |  |  |  | 0.866 | 0.794 | 0.946 |  |  |  |
| Without vocational training |  |  |  | 0.819 | 0.750 | 0.893 |  |  |  |
| With vocational training |  |  |  | 0.977 | 0.899 | 1.062 |  |  |  |
| Master craftsman/technician degree |  |  |  | 1.096 | 0.994 | 1.208 |  |  |  |
| College degree |  |  |  | 1(Ref.) |  |  |  |  |  |
| **Occupation** |  |  |  |  |  |  |  |  |  |
| Agriculture, forestry, animal husbandry and horticulture |  |  |  | 1(Ref.) |  |  |  |  |  |
| Raw material extraction, production and manufacturing |  |  |  | 1.055 | 0.989 | 1.125 |  |  |  |
| Construction, architecture, surveying and building services engineering |  |  |  | 1.055 | 0.985 | 1.130 |  |  |  |
| Natural science, geography and information technology |  |  |  | 1.147 | 1.042 | 1.262 |  |  |  |
| Transport, logistics, protection and security |  |  |  | 1.080 | 1.013 | 1.152 |  |  |  |
| Commercial services, goods trade, distribution, tourism |  |  |  | 1.221 | 1.139 | 1.310 |  |  |  |
| Business organization, accounting, law, administration |  |  |  | 1.456 | 1.357 | 1.563 |  |  |  |
| Health, social services, teaching and education |  |  |  | 1.186 | 1.107 | 1.270 |  |  |  |
| Language, literature, humanities, social and economic sciences, media, art, culture and design |  |  |  | 1.176 | 1.049 | 1.317 |  |  |  |
| Military |  |  |  | 2.019 | 0.959 | 4.251 |  |  |  |
| Position „Helper“ |  |  |  | 1(Ref.) |  |  |  |  |  |
| Position „Trained“ |  |  |  | 1.092 | 1.068 | 1.116 |  |  |  |
| Position „Specialist“ |  |  |  | 1.221 | 1.169 | 1.276 |  |  |  |
| Position „Management“ |  |  |  | 1.280 | 1.205 | 1.359 |  |  |  |
