# Supplementary material for: Indication for spinal surgery: associated factors and regional differences in Germany
Source: BMC Health Serv Res. 2022 Sep 1;22:1109. doi: 10.1186/s12913-022-08492-3 (PMC9438246; doi:10.1186/s12913-022-08492-3)
Supplement: Supplementary file 6 — Additional file 6. Extended description of cohort and subcohorts. [file 12913_2022_8492_MOESM6_ESM.docx]

**Supplementary Material**

Additional file 6: Sociodemographic, clinical, and medical care characteristics of cases/episodes with and without spinal surgery. For the cases with spinal surgery, the figures refer to the period before the corresponding hospitalization (in the period: 2008 to 2016). Since the 2010 activity code was only introduced in the social insurance system in November 2011, interventions for the subgroup of employees were only conducted for the years 2012 to 2016.

|  | All 2008-16 | | Employed 2012-16 | | Retired 2008-16 | |
| --- | --- | --- | --- | --- | --- | --- |
|  | All Cases | Cases with Spinal Surgery | All Cases | Cases with Spinal Surgery | All Cases | Cases with Spinal Surgery |
| **Sociodemographic** |  |  |  |  |  |  |
| Total | 60942755 | 444218 | 12195372 | 50668 | 23474885 | 226272 |
| Male | 24839868 | 195082 | 6145335 | 29273 | 7909135 | 82413 |
| Female | 35853751 | 249136 | 6050037 | 21395 | 15565750 | 143859 |
| Age group 0-19 | 1391462 | 3040 |  |  |  |  |
| Age group 20-24 | 1673525 | 2266 | 663989 | 515 |  |  |
| Age group 25-29 | 2174090 | 5139 | 951760 | 1468 |  |  |
| Age group 30-34 | 2487653 | 9050 | 1020821 | 2435 |  |  |
| Age group 35-39 | 3019274 | 14786 | 1069577 | 3437 |  |  |
| Age group 40-44 | 4219451 | 25491 | 1433565 | 6012 |  |  |
| Age group 45-49 | 5421768 | 35337 | 1954140 | 9271 |  |  |
| Age group 50-54 | 5896338 | 40720 | 2127035 | 11396 |  |  |
| Age group 55-59 | 5848896 | 42756 | 1832971 | 10464 |  |  |
| Age group 60-64 | 5176657 | 38453 | 1141514 | 5670 |  |  |
| Age group 65-69 | 4698260 | 47898 |  |  | 4538882 | 47346 |
| Age group 70-74 | 5861838 | 68654 |  |  | 5762474 | 68417 |
| Age group 75-79 | 5635845 | 61994 |  |  | 5623684 | 61889 |
| Age group 80-84 | 4097102 | 33767 |  |  | 4094320 | 33756 |
| Age group 85-89 | 2351340 | 12453 |  |  | 2350640 | 12451 |
| Age group 90-94 | 793966 | 2196 |  |  | 791645 | 2195 |
| Age group 95+ | 195290 | 218 |  |  | 195064 | 218 |
| **Comorbidities** |  |  |  |  |  |  |
| Osteoarthritis (knee) none | 50326119 | 362819 | 11449250 | 46894 | 16111407 | 164498 |
| Osteoarthritis (knee) | 10616636 | 81399 | 742348 | 3774 | 7363478 | 61774 |
| Osteoarthritis (hip) none | 54294967 | 388596 | 11815863 | 48497 | 18575525 | 182909 |
| Osteoarthritis (hip) | 6647788 | 55622 | 379509 | 2171 | 4899360 | 43363 |
| Osteoporosis none | 54683170 | 389228 | 12029943 | 49851 | 18259763 | 178317 |
| Osteoporosis | 6259585 | 54990 | 165429 | 817 | 5215122 | 47955 |
| Chronic rheumatoid polyarthritis none | 59005153 | 439185 | 12077039 | 49627 | 22307647 | 212678 |
| Chronic rheumatoid polyarthritis | 1937602 | 19579 | 169065 | 1041 | 1167238 | 13594 |
| Other rheumatic diseases with typical spine involvement none | 60226032 | 424639 | 12110866 | 50332 | 23205182 | 223724 |
| Other rheumatic diseases with typical spine involvement | 716723 | 5033 | 84506 | 336 | 269703 | 2548 |
| Other rheumatic diseases without typical spine involvement none | 59301709 | 431852 | 12026307 | 50137 | 22266007 | 215999 |
| Other rheumatic diseases without typical spine involvement | 1641046 | 12366 | 118333 | 531 | 1208878 | 10273 |
|  |  |  |  |  |  |  |
| Depression none | 48577608 | 358239 | 10616094 | 43702 | 17922739 | 178791 |
| Depression | 12365147 | 85979 | 1579278 | 6966 | 5552146 | 47481 |
| Anxiety disorder none | 57203407 | 424504 | 11647539 | 48803 | 22165526 | 216662 |
| Anxiety disorder | 3739348 | 19714 | 547833 | 1865 | 1309359 | 9610 |
| Psychosomatic disorders none | 53319022 | 397193 | 11075795 | 46288 | 20412184 | 201432 |
| Psychosomatic disorders | 7623733 | 47025 | 1119577 | 4380 | 3062701 | 24840 |
| Sleep disorders none | 55064869 | 397364 | 11546329 | 47497 | 20110543 | 195057 |
| Sleep disorders | 5877886 | 46854 | 598375 | 3171 | 3364342 | 31215 |
| Dementia none | 58793909 | 435816 |  |  | 21461138 | 218376 |
| Dementia | 2148846 | 8402 |  |  | 2013747 | 7896 |
| **Physician consultations** |  |  |  |  |  |  |
| General practitioner | 26987138 | 110763 | 6250385 | 12056 | 10589280 | 60587 |
| One orthopedic specialist | 16554747 | 127586 | 2695065 | 13802 | 6332497 | 65906 |
| General practitioner and one orthopedic specialist | 7390065 | 29619 | 1300235 | 2551 | 2851103 | 17497 |
| One neurosurgeon without involvement of orthopedic specialist | 870269 | 26929 | 175702 | 4202 | 293434 | 11501 |
| Several orthopedic specialist neurosurgeons | 5434712 | 20373 | 1171199 | 2030 | 2109631 | 11918 |
| No involvement of general practitioner orthopedic specialist neurosurgeon | 3705824 | 128948 | 586433 | 16027 | 1298940 | 58863 |
| **Imaging of the spine** |  |  |  |  |  |  |
| MRI none | 52672125 | 221526 | 10492206 | 20401 | 20917694 | 127778 |
| MRI 1 | 6919339 | 161209 | 1450867 | 21284 | 2131479 | 74562 |
| MRI 2+ | 1351291 | 61483 | 252299 | 8983 | 425712 | 23932 |
| CT none | 57289426 | 349520 | 11772978 | 42583 | 20917694 | 177556 |
| CT 1 | 3233975 | 79593 | 383700 | 7039 | 2131479 | 40972 |
| CT 2+ | 419354 | 15105 | 38694 | 1046 | 42571 | 7744 |
| X-ray none | 40991848 | 209763 | 9002601 | 25621 | 21875790 | 108090 |
| X-ray 1 | 1635088 | 164243 | 2734607 | 8350 | 1393132 | 2366 |
| X-ray 2+ | 3600018 | 70212 | 458164 | 6697 | 205963 | 35816 |
| Myelography/ Electroneurography no | 60399719 | 428592 | 12142135 | 49453 | 16060914 | 217481 |
| Myelography/ Electroneurography 1 | 393200 | 14435 | 38116 | 1148 | 6021130 | 8091 |
| Myelography/ Electroneurography 2+ | 149836 | 1191 | 15121 | 67 | 1392841 | 700 |
| **Pain medication** |  |  |  |  |  |  |
| NSAID none | 2280175 | 86632 | 5033213 | 8360 | 8676299 | 48291 |
| NSAID 0.1 to 30 DDD | 1492378 | 75653 | 3570600 | 10120 | 4445625 | 33963 |
| NSAID 30 to 90 DDD | 1237895 | 110502 | 2434349 | 15922 | 4594837 | 48478 |
| NSAID 90 to 180 DDD | 5159759 | 67350 | 699586 | 8324 | 2477666 | 33347 |
| NSAID 180+ DDD | 5678512 | 104081 | 457624 | 7942 | 3280458 | 62193 |
| Cox-2 inhibitors none | 5739116 | 391117 | 11679878 | 44599 | 21730373 | 197757 |
| Cox-2 inhibitors 0.1 to 30 DDD | 1749164 | 15395 | 291688 | 1665 | 784236 | 8006 |
| Cox-2 inhibitors 30 to 90 DDD | 844318 | 19722 | 125685 | 2708 | 423240 | 10310 |
| Cox-2 inhibitors 90 to 180 DDD | 436812 | 7430 | 52153 | 792 | 236691 | 4113 |
| Cox-2 inhibitors 180+ DDD | 521301 | 10554 | 45968 | 904 | 300345 | 6086 |
| Non-opioid analgesics none | 4194065 | 215235 | 9469213 | 5892 | 14315520 | 100366 |
| Non-opioid analgesics 0.1 to 30 DDD | 14702036 | 154374 | 2480903 | 19713 | 6261009 | 78278 |
| Non-opioid analgesics 30 to 90 DDD | 275955 | 48071 | 196372 | 3921 | 1767737 | 29967 |
| Non-opioid analgesics 90 to 180 DDD | 86943 | 14904 | 32027 | 753 | 634050 | 9962 |
| Non-opioid analgesics 180+ DDD | 67108 | 11634 | 16857 | 389 | 496569 | 7699 |
| weak-acting opioids none | 52499761 | 288050 | 11425752 | 36047 | 18655790 | 139464 |
| weak-acting opioids 0.1 to 30 DDD | 4422395 | 78778 | 561769 | 9317 | 2168686 | 39724 |
| weak-acting opioids 30 to 90 DDD | 1721148 | 37011 | 117993 | 3219 | 1082126 | 21390 |
| weak-acting opioids 90 to 180 DDD | 749522 | 13698 | 32702 | 826 | 520343 | 8633 |
| weak-acting opioids 180+ DDD | 1549929 | 26681 | 57156 | 1259 | 1047940 | 17061 |
| strong-acting opioids none | 58359231 | 87144 | 2099682 | 47771 | 21632242 | 188876 |
| strong-acting opioids 0.1 to 30 DDD | 982742 | 25665 | 59181 | 1805 | 68156 | 16854 |
| strong-acting opioids 30 to 90 DDD | 423858 | 9635 | 13299 | 448 | 320020 | 6654 |
| strong-acting opioids 90 to 180 DDD | 308740 | 5411 | 7183 | 211 | 237683 | 3742 |
| strong-acting opioids 180+ DDD | 86818 | 16363 | 16027 | 433 | 603377 | 10146 |
| **Pain therapy** |  |  |  |  |  |  |
| Pain therapy care: none | 59770780 | 419109 | 12040322 | 47963 | 22966915 | 213587 |
| Pain therapy care: 1 | 441445 | 10040 | 71471 | 1278 | 177077 | 4863 |
| Pain therapy care: 2+ | 730530 | 15069 | 83579 | 1427 | 330893 | 7822 |
| Spinal manipulative therapy keine | 42226682 | 269009 | 8489986 | 27648 | 17847959 | 148440 |
| Spinal manipulative therapy 1 | 9501417 | 68226 | 2037339 | 9030 | 2710054 | 30675 |
| Spinal manipulative therapy 2+ | 9214656 | 106983 | 1668047 | 13990 | 2916872 | 47157 |
| Acupuncture: none | 56120755 | 377509 | 11556108 | 44252 | 21296295 | 189397 |
| Acupuncture: till 10 sessions | 3614461 | 48016 | 514851 | 5043 | 1540327 | 25198 |
| Acupuncture: 11+sessions | 1207539 | 18693 | 124413 | 1373 | 638263 | 11677 |
| Multimodal pain therapy none | 60752445 | 436814 | 12169071 | 49846 | 23399315 | 222946 |
| Multimodal pain therapy | 190310 | 7404 | 26301 | 822 | 75570 | 3326 |
| Injection therapy none | 54917291 | 307504 | 11346444 | 35141 | 20768597 | 157907 |
| Injection therapy 1 | 2996389 | 45469 | 469087 | 5384 | 1265711 | 22579 |
| Injection therapy 2+ | 3029075 | 91245 | 379841 | 10143 | 1440577 | 45786 |
| **Physical Therapy (Indication Spine)** |  |  |  |  |  |  |
| Exercise therapy none | 46250127 | 279676 | 9461263 | 28580 | 18061418 | 145342 |
| Exercise therapy 1 Prescription | 8054659 | 73535 | 1601882 | 9788 | 2733875 | 35027 |
| Exercise therapy 2 Prescription | 3287757 | 38717 | 601285 | 5438 | 1243612 | 18957 |
| Exercise therapy 3-5 Prescription | 2603092 | 39338 | 441884 | 5465 | 1072919 | 19958 |
| Exercise therapy 6+ Prescription | 747120 | 12952 | 89058 | 1397 | 363061 | 6988 |
| Manual therapy none | 54401969 | 383108 | 10685304 | 41245 | 21174288 | 196713 |
| Manual therapy 1 Prescription | 3767685 | 31105 | 895773 | 4859 | 1251588 | 14529 |
| Manual therapy 2 Prescription | 1469170 | 14074 | 335587 | 2182 | 535668 | 6904 |
| Manual therapy 3-5 Prescription | 1066576 | 12490 | 235839 | 1908 | 413664 | 6365 |
| Manual therapy 6+ Prescription | 237355 | 3441 | 42869 | 474 | 99677 | 1761 |
| Massage therapy none | 52896412 | 381878 | 11086685 | 45074 | 20064705 | 192414 |
| Massage therapy 1 Prescription | 5175196 | 38608 | 793718 | 3810 | 2060474 | 20328 |
| Massage therapy 2 Prescription | 1724473 | 13740 | 209982 | 1117 | 782836 | 7755 |
| Massage therapy 3-5 Prescription | 986291 | 8445 | 95484 | 608 | 483597 | 4892 |
| Massage therapy 6+ Prescription | 160383 | 1547 | 9503 | 59 | 83273 | 883 |
| **Rehabilitation/Needed care** |  |  |  |  |  |  |
| inpatient rehabilitation none |  |  |  |  | 23422380 | 224874 |
| inpatient rehabilitation |  |  |  |  | 52505 | 1398 |
| needed care none |  |  |  |  | 19570875 | 206062 |
| Needed care stage I |  |  |  |  | 245227 | 16008 |
| Needed care stage II |  |  |  |  | 1178783 | 3981 |
| Needed care stage III |  |  |  |  | 272953 | 221 |
| **Sick leave days** |  |  |  |  |  |  |
| none |  |  | 6801063 | 2430 |  |  |
| 1-7 days |  |  | 1959149 | 998 |  |  |
| 8-21 days |  |  | 1729822 | 1604 |  |  |
| 22-42 days |  |  | 805911 | 2364 |  |  |
| 42+ days |  |  | 899427 | 43272 |  |  |
| **Education** |  |  |  |  |  |  |
| Unknown school-leaving qualification |  |  | 4160607 | 17262 |  |  |
| No school-leaving qualification |  |  | 283132 | 1211 |  |  |
| Lower Secondary leaving certificate |  |  | 3625250 | 18800 |  |  |
| Intermediate school leaving certificate |  |  | 3107521 | 11207 |  |  |
| High school diploma |  |  | 1018862 | 2188 |  |  |
| Unknown vocational training |  |  | 2451052 | 9607 |  |  |
| Without vocational training |  |  | 1965000 | 8230 |  |  |
| With vocational training |  |  | 7032560 | 30817 |  |  |
| Master craftsman/technician degree |  |  | 349603 | 1245 |  |  |
| College degree |  |  | 397157 | 769 |  |  |
| **Occupation** |  |  |  |  |  |  |
| Agriculture forestry animal husbandry and horticulture |  |  | 254651 | 1008 |  |  |
| Raw material extraction production and manufacturing |  |  | 3315982 | 15217 |  |  |
| Construction architecture surveying and building services engineering |  |  | 903911 | 4710 |  |  |
| Natural science geography and information technology |  |  | 209137 | 737 |  |  |
| Transport logistics protection and security |  |  | 2659743 | 12969 |  |  |
| Commercial services goods trade distribution tourism |  |  | 1374728 | 4690 |  |  |
| Business organization accounting law administration |  |  | 1447752 | 4392 |  |  |
| Health social services teaching and education |  |  | 1872707 | 6502 |  |  |
| Language literature humanities social and economic sciences media art culture and design |  |  | 155920 | 436 |  |  |
| Military |  |  | 840 | 7 |  |  |
| Position „Helper“ |  |  |  |  |  |  |
| Position „Trained“ |  |  | 3225968 | 14046 |  |  |
| Position „Specialist“ |  |  | 7640283 | 32510 |  |  |
| Position „Management“ |  |  | 854911 | 2772 |  |  |
